# Supplementary figures and images for: Canonical correlation analysis for multi-omics: Application to cross-cohort analysis
Source: PLoS Genet. 2023 May 22;19(5):e1010517. doi: 10.1371/journal.pgen.1010517 (PMC10237647; doi:10.1371/journal.pgen.1010517)

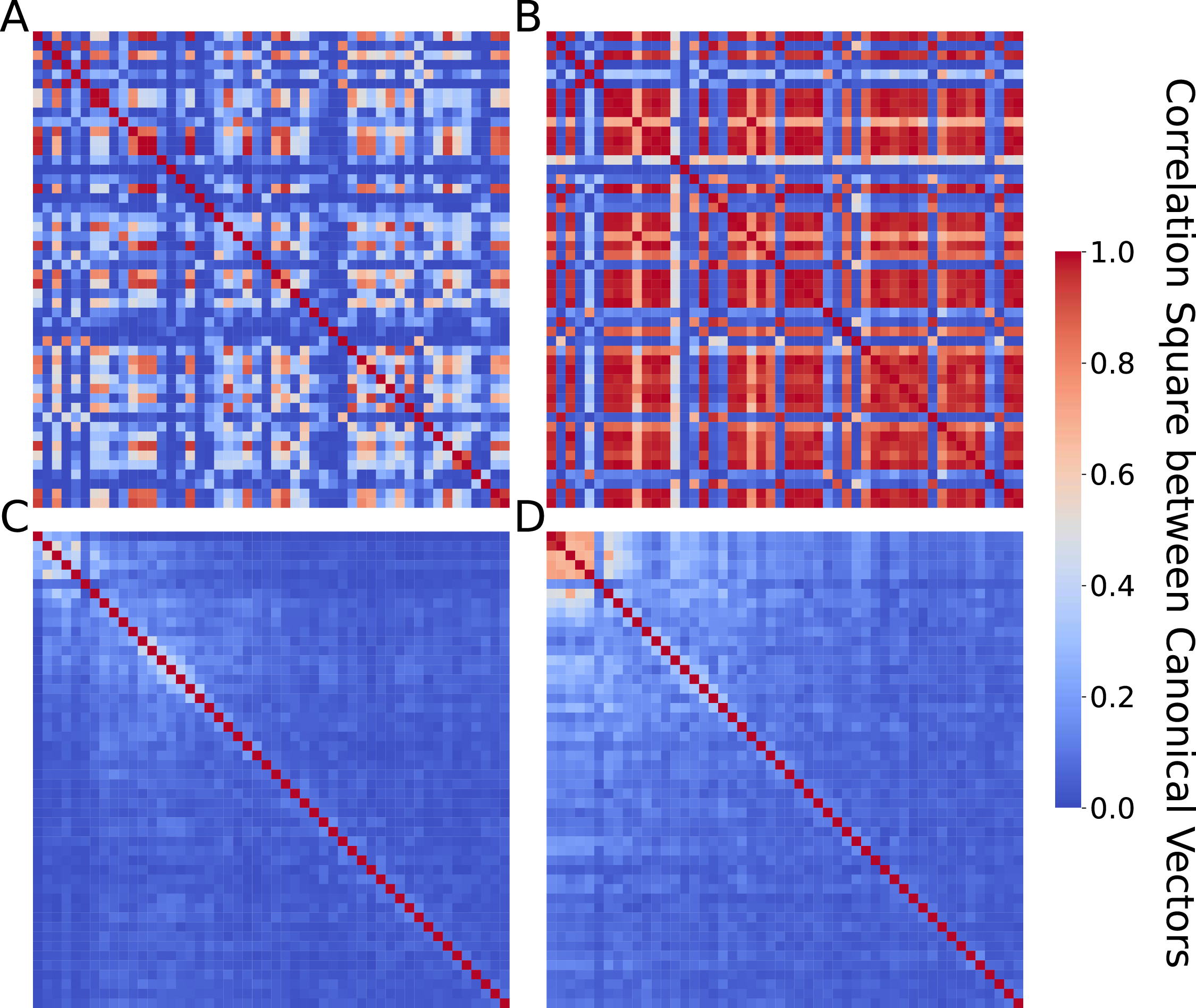

Supplement: S1 Fig — CVs are inferred from JHS proteomics and methylomics data using unsupervised SMCCA. Each row and column represent one CV, ranging from CV1 to CV50. (A-B) Results from the PMA package, implementation of the original SMCCA methods without the incorporation of GS algorithm. (C-D) Results from our SMCCA-GS, with the GS strategy incorporated. Left panel (A and C) show proteomics CVs, and right panel (B and D) from methylomics CVs. (TIF) [file pgen.1010517.s001.tif]

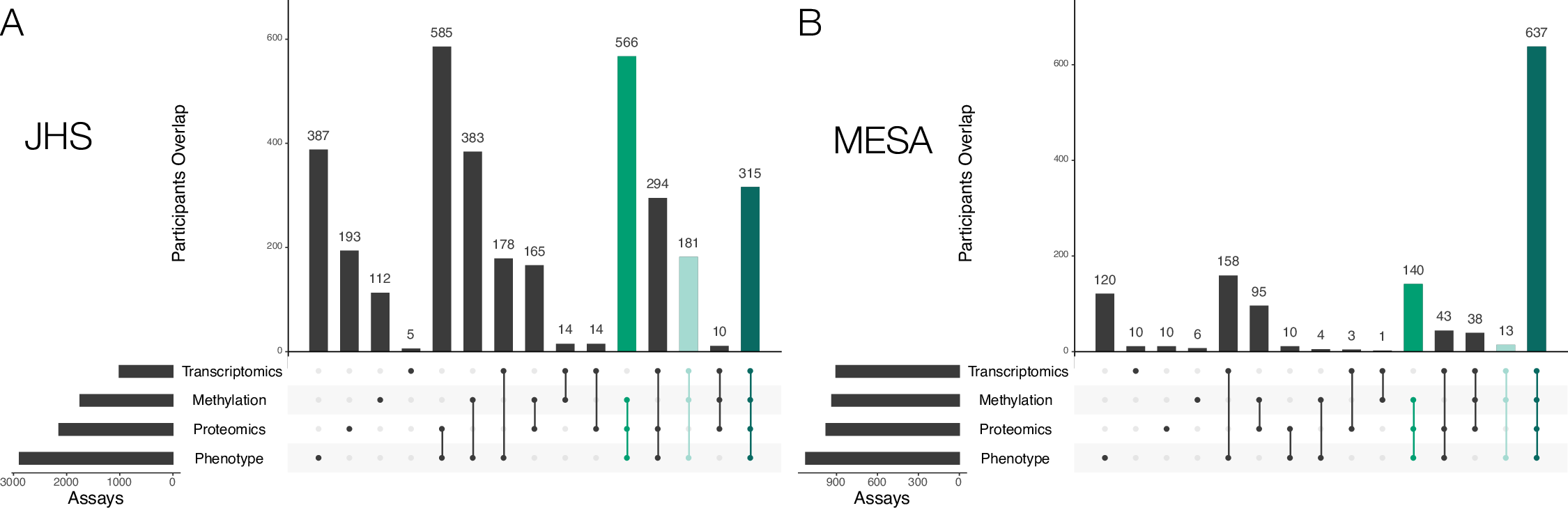

Supplement: S2 Fig — (A) JHS: 881 participants have complete proteomics, methylation, and phenotype information; 496 participants have complete transcriptomics, methylation, and phenotype information. (B) MESA: 777 participants have complete proteomics, methylation, and phenotype information; 650 participants have complete transcriptomics, methylation, and phenotype information. (TIF) [file pgen.1010517.s002.tif]

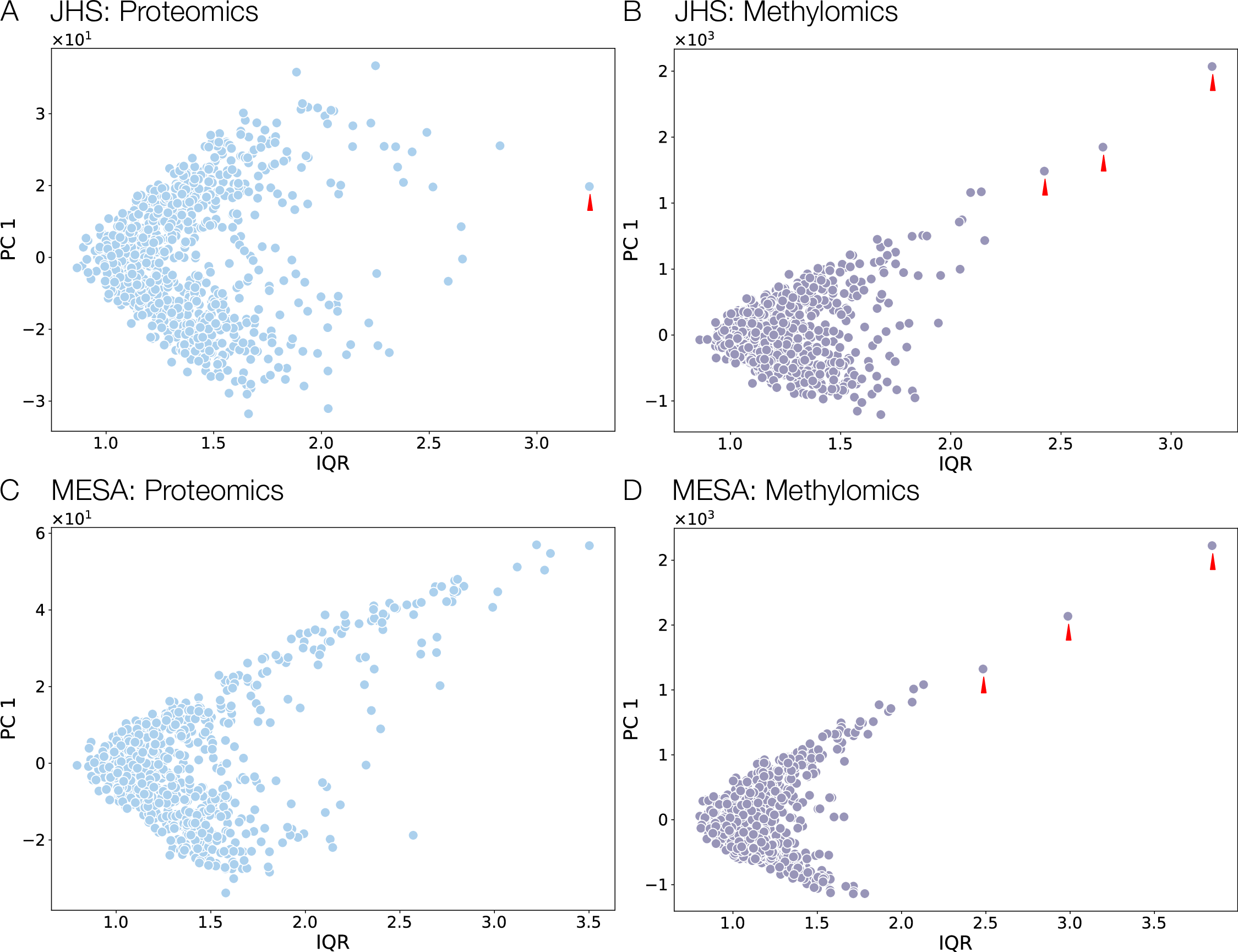

Supplement: S3 Fig — Each dot in the plot represents one individual. X-axis is the interquartile range (IQR) while Y-axis is the top principal component (PC). (A) JHS proteomics: one outlier was detected, marked by the wedge pointer; (B) JHS methylomics: three outliers were detected; (C) MESA proteomics: MESA: no outliers; (D) MESA methylomic: three outliers were detected. (TIF) [file pgen.1010517.s003.tif]

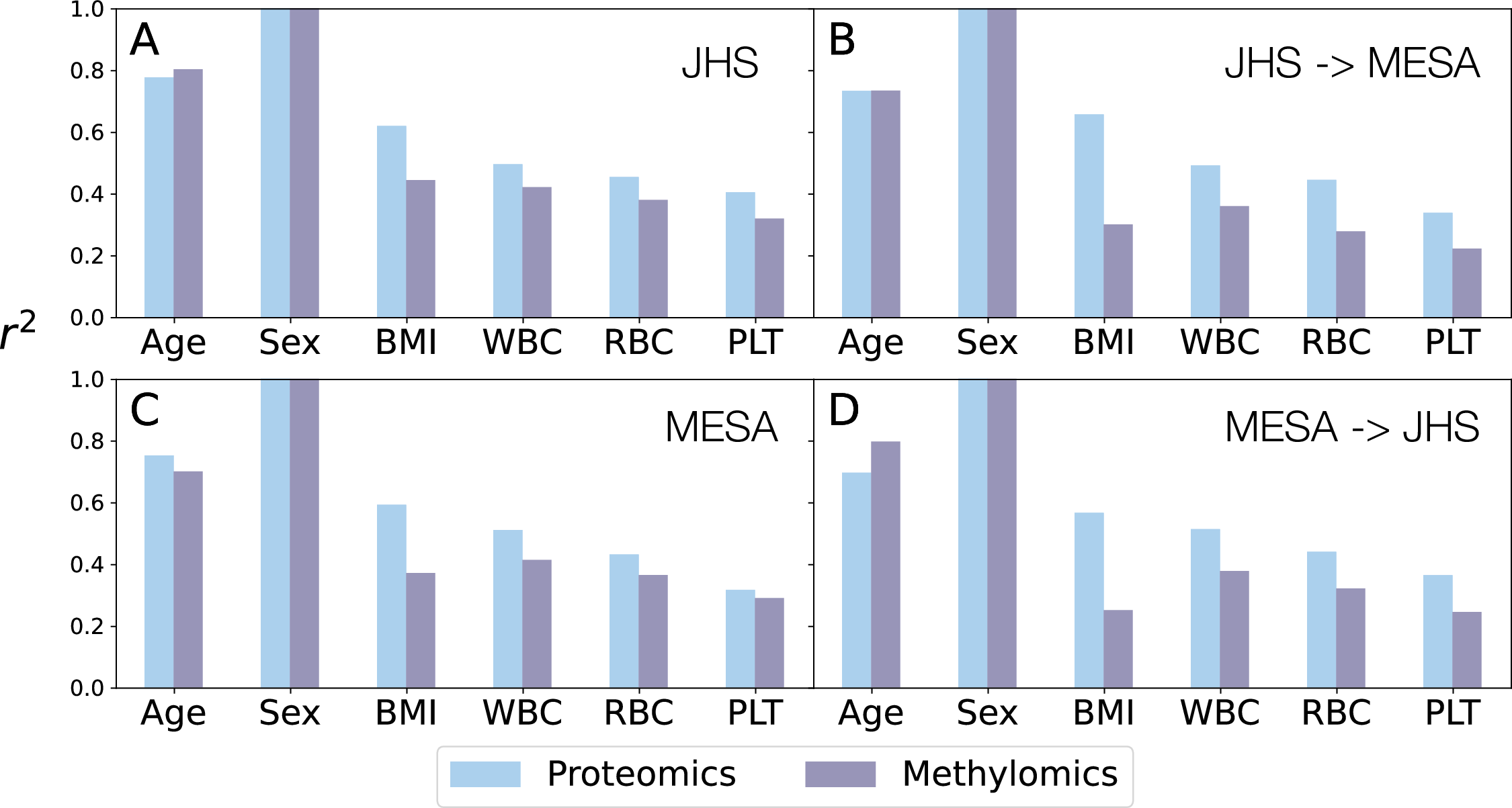

Supplement: S4 Fig — (A) CVs were inferred using proteomics and all ~700k CpG sites in JHS. The top 50 CVs were used to calculate the r2 (Y-axis) for each outcome (X-axis). (B) We obtained CVs in JHS by applying the weights inferred from MESA, and then calculated r2 in the same way as in A. (C) CVs were inferred using proteomics and all ~700k CpG sites in MESA. (D) CVs were obtained in MESA by applying the weights inferred from JHS. (TIF) [file pgen.1010517.s004.tif]

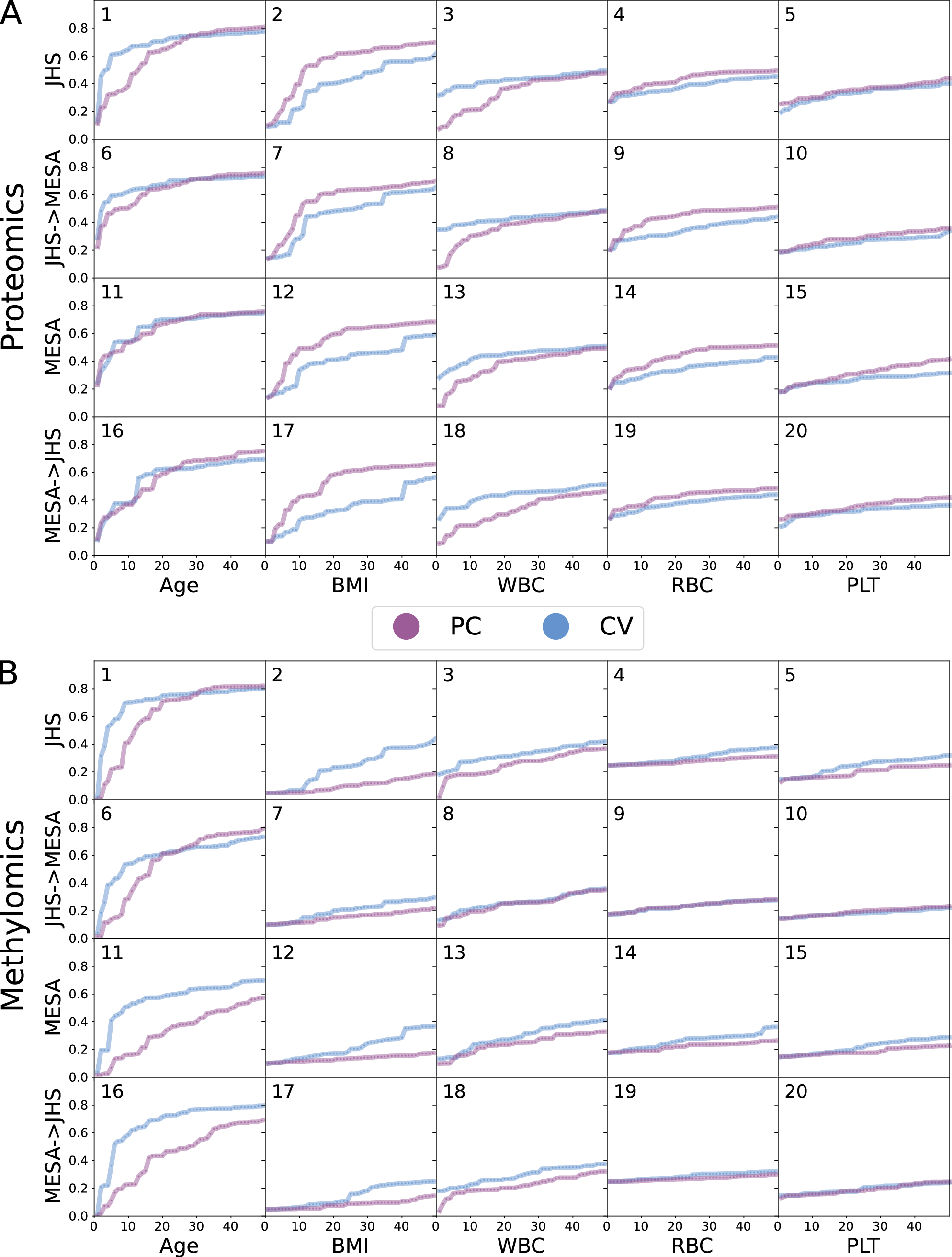

Supplement: S5 Fig — Each column is for one outcome. Top row (JHS) shows results in JHS using JHS-inferred CVs. Second row (JHS->MESA) shows results in MESA, also using JHS-inferred CV weights. Third row (MESA) shows results in MESA, this time using MESA-inferred CVs. Last row (MESA->JHS) shows results in JHS, also using MESA-inferred CV weights. (A) Proteomics. (B) Methylomics. In each sub-figure, X-axis indicates the number of CVs or PCs used and Y-axis the proportion of variation explained in the (i.e., r2). (TIF) [file pgen.1010517.s005.tif]

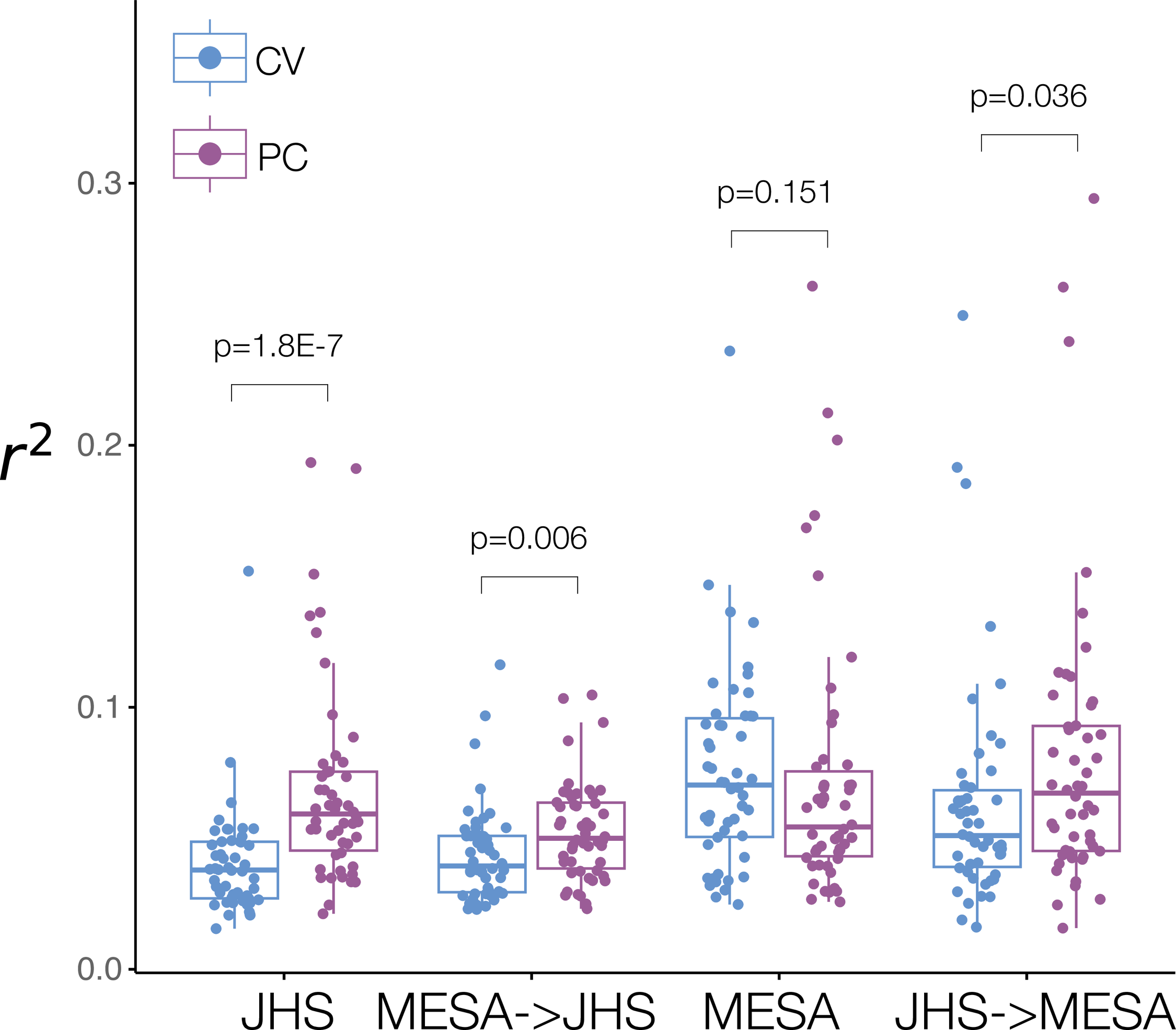

Supplement: S6 Fig — For JHS, the proteomics technical variable is batch-plate combination status. For MESA, the proteomics technical variable is plate. (TIF) [file pgen.1010517.s006.tif]

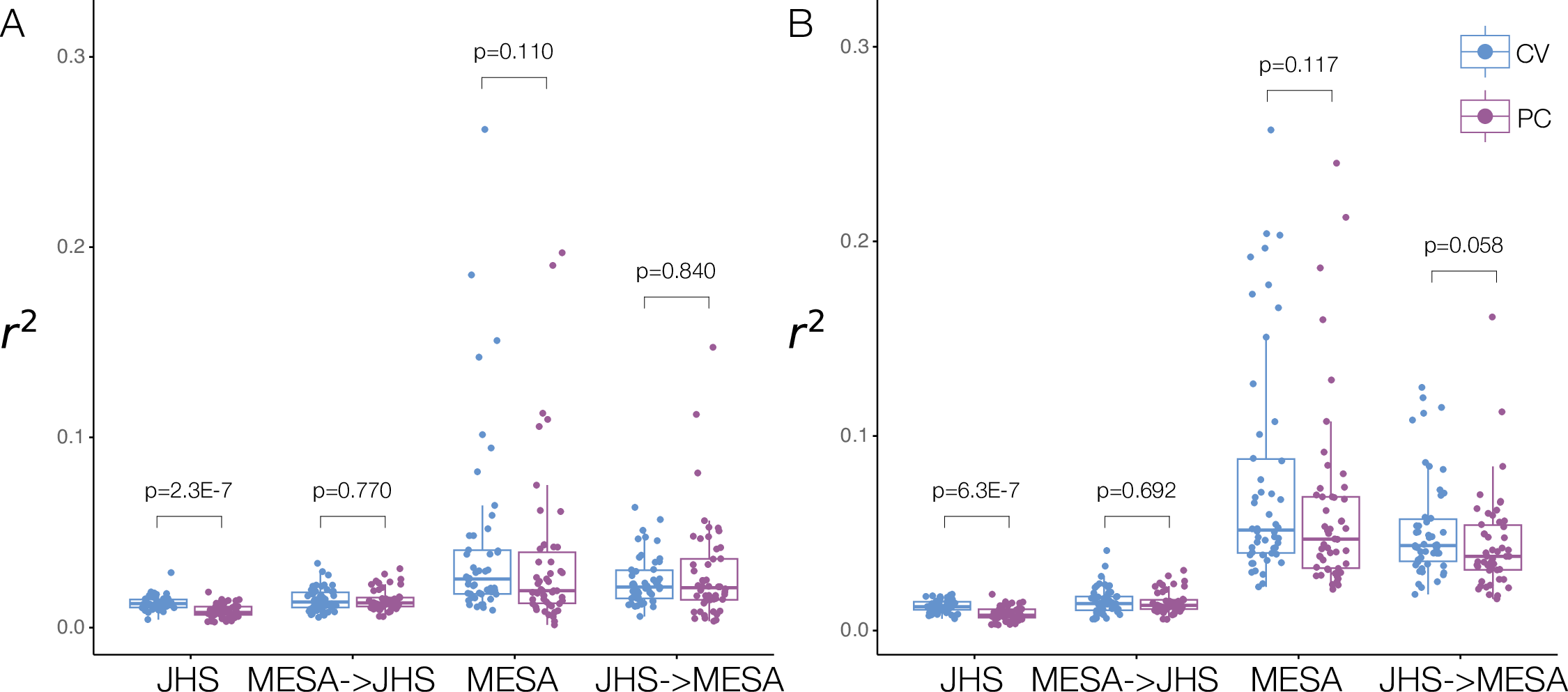

Supplement: S7 Fig — For JHS, the methylomics technical variable is group-plate combination status. For MESA, the methylomics technical variables are (A) “Batch Scan”, and (B) “Level1 Batch”. (TIF) [file pgen.1010517.s007.tif]
