# Supplementary material for: Canonical correlation analysis for multi-omics: Application to cross-cohort analysis
Source: PLoS Genet. 2023 May 22;19(5):e1010517. doi: 10.1371/journal.pgen.1010517 (PMC10237647; doi:10.1371/journal.pgen.1010517)
Supplement: S1 Text — (PDF) [file pgen.1010517.s016.pdf]

S1 Text. Supplementary Information.

Section 1

SMCCA proposed by Witten et al. (2009) [1] serves as the backbone of our methods. The objective function is the same as in the original SMCCA paper, detailed in their section 3 “Sparse multiple CCA”, and their original algorithm was implemented in the Multi.CCA and Multi.CCA.permute functions in the PMA R package. For penalty, we adopt the same algorithm implemented in the Multi.CCA function, which uses the L1 penalty and requires a parameter tuned in the following way.

1. For each assay, we initiate the following list of parameter candidates as coded in the PMA R package: (max(0.1 x sqrt(k), 1.1), max(0.178 x sqrt(k), 1.1), max(0.256 x sqrt(k), 1.1), max(0.333 x sqrt(k), 1.1), max(0.411 x sqrt(k), 1.1), max(0.489 x sqrt(k), 1.1), max(0.567 x sqrt(k), 1.1), max(0.644 x sqrt(k), 1.1), max(0.722 x sqrt(k), 1.1), max(0.8 x sqrt(k), 1.1)), where k is the number of features in the assay.

2. For the 1st parameter candidate (that is, parameter candidate index $j = 1$) in the list above, we run SMCCA with each candidate to calculate CVs and the across-assay sum correlation.

3. We then randomly permute each assay and re-run SMCCA with the same parameter candidate to calculate the sum correlation for the permuted assay. We compare this sum correlation with the sum correlation obtained in step 2.

4. For the same parameter, we repeat step 3 for a default of 10 times and obtain N_1, which is the number of permutations where the sum correlation is larger than the sum correlation obtained in step 2, where 1 is the index for the parameter in the list of parameter candidates.

5. For each of the other parameter candidates, we carry out steps 2-4 above and obtain the N_j’s (again, j is the parameter candidate index) and select the one with the largest N_j as the optimal parameter.

For our SMCCA-GS method, we modified the original SMCCA algorithm to implement the GS algorithm. Specifically, we update the input assays fed to the original SMCCA functions and repeat the inference process every time after we obtain a new set of CVs, in a sequential manner. The algorithm is described in **Algorithm 2**.

Algorithm 2 SMCCA-GS

Input: $\{\mathbf{X_{i,1}}\}_{i=1,\ldots,N}$ ▷ N assays, subscript $_{i,1}$ denotes the original ith assay

for $k \leftarrow 1$ to K **do** ▷ Compute K CVs
  $\mathbf{CV_{i,k}}, \mathbf{w_{i,k}} \leftarrow \texttt{SMCCA}(\{\mathbf{X_{i,k}}\})$ ▷ `SMCCA` from **PMA** R Package ▷ $\mathbf{CV_{i,k}}, \mathbf{w_{i,k}}$ are kth CV and weight for the ith assay
  for $i \leftarrow 1$ to N **do**
    $\mathbf{X_{i,k+1}} \leftarrow \mathbf{X_{i,k}} - \mathbf{CV_{i,k}}\mathbf{w_{i,k}}^\mathsf{T}$ ▷ Update input assays by removing the effects of current CVs
  end for
end for

Output: $\{\mathbf{CV_{i,k}}\}_{i=1,\ldots,N,k=1,\ldots,K}$ ▷ K CVs for each assay

Section 2

We detect sample outliers in the following way.

1. Compute the first principal component (PC) and interquartile range (IQR) of an assay and standardize both the PC and IQR.
2. Compute the centroid on the standardized PCA-IQR plot and the distance from each individual to the centroid. Calculate the mean distance ($\overline{d_c}$) and standard deviation (σ_{d_c}) of the distances. Generate the list of centroid-based outlier candidates by selecting individuals whose distances to the centroid exceed a threshold value $\overline{d_c} + k \cdot \sigma_{d_c}$, where we empirically set $k = 6$.
3. Calculate the distance of each individual on the standardized PCA-IQR plot to its closed neighboring individual (defined by the smallest Euclidean distance), naming it the closest-neighbor-distance. Determine the mean ($\overline{d_d}$) and standard deviation (σ_{d_d}) of the closest-neighbor-distances. Build the list of closest-neighbor-distance-based outlier candidates by selecting individuals whose closest-neighbor-distances exceed a threshold value $\overline{d_d} + k \cdot \sigma_{d_d}$, where again we empirically set $k = 6$.
4. The final outliers are obtained by intersecting the two lists of outlier candidates.

Reference

1. Witten DM, Tibshirani RJ. Extensions of sparse canonical correlation analysis with applications to genomic data. Stat Appl Genet Mol Biol. 2009;8: Article28.
