# Supplementary material for: Canonical correlation analysis for multi-omics: Application to cross-cohort analysis
Source: PLoS Genet. 2023 May 22;19(5):e1010517. doi: 10.1371/journal.pgen.1010517 (PMC10237647; doi:10.1371/journal.pgen.1010517)
Supplement: S1 Acknowledgement — (PDF) [file pgen.1010517.s017.pdf]

NHLBI Trans-Omics for Precision Medicine (TOPMed) Consortium

Claire Hill, Alana Jones, Gonçalo Abecasis, Avinash Abhyankar, Mohit Aggarwal, Francois Aguet, Rachel Akers, Babatunde Akinwunmi, Elie Akl, Rafet Al-Tobasei, Christine Albert, Micheala Aldred, Rama Alhariri, Taryn Alkis, Laura Almasy, Marcio Almeida, Alvaro Alonso, Meghan Althoff, Seth Ament, Farah Ammous, Elizabeth Ampleford, Ping An, Christopher D. Anderson, Charlotte Andersson, Kurtis Anthony, Pramod Anugu, Milla Arabadjian, Kristin Ardlie, Dan Arking, Sebastian M. Armasu, Donna K Arnett, Pankaj Arora, Heather Arruda, Marios Arvanitis, Allison Ashley-Koch, Aneel Ashrani, Stella Aslibekyan, Tim Assimes, Elizabeth Atkinson, Paul Auer, Thomas R. Austin, Christy Avery, Julian Avila-Pacheco, Paul Avillach, Abraham Aviv, Dimitrios Avramopoulos, Najib Ayas, Andrea Baccarelli, Minoo Bagheri, Ravi Balijepalli, Christie Ballantyne, Pallavi Balte, Michael Bamshad, Farnoush Banaei-Kashani, Mike Bancks, Wei Bao, Jacob Barber, Andree-Ann Baril, John Barnard, Kathleen Barnes, R. Graham Barr, Emily Barron-Casella, Traci Bartz, Saonli Basu, Alexis Battle, Stephanie Battle, Terri Beaty, Andrew Beaudin, Gerald Beck, Lewis Becker, Diane Becker, Ferdouse Begum, Alexa Beiser, Amber Beitelshees, Reza Bekheirnia, Emelia Benjamin, Takis Benos, Hanna Berk-Rauch, Zachary M Besich, Marcos Bezerra, Surya Bhatt, Romit Bhattacharya, Alexander Bick, Larry Bielak, Mary Lou Biggs, Joshua Bis, Thomas Blackwell, John Blangero, Eugene Bleecker, Elizabeth Blue, Nathan Blue, Corneliu Bodea, Mike Boehnke, Eric Boerwinkle, Kelly Bolton, Jessica Bon, Silvia Bonas, Christine Borchert, Victor Borda, Donald W. Bowden, Joel Bowen, Russell Bowler, Harrison Brand, Ashley Brant, Jan Bressler, Jennifer Brody, Ulrich Broeckel, Matthew Brown, Michael Brown, Derek Brown, Deborah Brown, Brielin Brown, Sharon Browning, Brian Browning, Martina Brueckner, Esteban Burchard, Robert Burk, Deepika Burkardt, Kristina Buschur, Carlos Bustamante, Javed Butler, Brian Cade, Jennifer Caldwell, Sarah Calvo, Jonathan Cardwell, Vincent Carey, Jenna Carlson, April P. Carson, Cara Carty, Richard Casaburi, James Casella, Andrea Cassar, Peter Castaldi, Christina A Castellani, Olga Cecilia Castro Diehl, Liam Cato, Katie Cederberg, Dima Chaar, Mark Chaffin, Mark Chaisson, Aravinda Chakravarti, Nathalie Chami, Kei Hang Katie Chan, Mark Chandy, I-Shou Chang, Christy Chang, Yi-Cheng Chang, Sinead Chapman, Daniel Chasman, Nilanjan Chatterjee, Sameer Chavan, Ming-Huei Chen, Han Chen, Zhanghua Chen, Zsu-Zsu Chen, Wei-Min Chen, Shu Hui Chen, Yii-Der Ida Chen, Chung-Shiuan Chen, Feixiong Cheng, Susan Cheng, Yen-Feng Chiu, Michael Cho, So Mi Cho, Seung Hoan Choi, Jessica Chong, Zechen Chong, David C. Christiani, Ingrid Elisabeth Christophersen, Lee-Ming Chuang, Sumeet Chugh, Jaeyoon Chung, Ren-Hua Chung, Mina Chung, Mina Cintho Ozahata, Mete Civelek, Steven Claas, Robert Clark, Shoa Clarke, Danielle Clarkson-Townsend, Melina Claussnitzer, Jean Rene Clemenceau, Melissa Cline, Clary Clish, Sean Coady, Zeynep Coban Akdemir, Bradley Coe, John Cole, Jason Collins, Ryan Collins, Claudia Colmenares, Suzy Comhair, Karen Conneely, Matthew Conomos, Brandon Coombes, Matthew Cordes, Josef Coresh, Laura Corlin, Elaine Cornell, André Corvelo, Paul Courchesne, Laura Courtney, Carolyn Crandall, James Crapo, Damien Croteau-Chonka, Dan Cruz, L. Adrienne Cupples, Joanne Curran, Brian Custer, Michael Cutler, Yang Dai, Coleen Damcott, Scott Damrauer, Reese Danaceau, Dawood Darbar, Chandra Dass, Sean David, Cutler David, Nicole Davis Armstrong, Warwick Daw, Michelle Daya, Mariza de Andrade, Luciana De Brito Vargas, Lisa de las Fuentes, Paul de Vries, Michael DeBaun, Thomas W. DeCato, Christopher Defilippi, Ranjan Deka, Chris Delaney, Tracie DeLuca, Dawn DeMeo, Serkalem Demissie, Shuliang Deng, Xutao Deng, Rajat Deo, Vimal Derebail, Pinkal Desai, Anita DeStefano, Kacie Deters, Scott Devine, Ana F. Diallo, Daniel DiCorpo, Katarina DiLillo, Carla Dinardo, Jingzhong Ding, Jun Ding, Jasmin Divers, Avantika Diwadkar, Ron Do, Harsha Doddapaneni, Arce Domingo-Relloso, Weilai Dong, Huawei Dong, Alessandro Doria, Jacqueline Dron, Shiron Drusinsky, Emily Morgan Drzymalla, Margaret Mengmeng Du, Zhaohui Du, Mulong Du, Qing Duan, Ruth Dubin, Shannon Dugan-Perez, Priya

Duggal, Ravi Duggirala, Andrea Dunaif, Vy Duong, Josée Dupuis, Jon Peter Durda, Susan K. Dutcher, Joyita Dutta, Eric Earley, Charles Eaton, Walter Eckalbar, Todd Edwards, Clara Ehrman, Evan Eichler, Aaron Eisman, Lynette Ekunwe, Adel El Boueiz, Patrick Ellinor, Amanda Elliott, Connor Emdin, Celeste Eng, Zeynep Erdoğan-Yıldırım, Serpil Erzurum, Tonu Esko, Luke Evans, Fang Fang, Nauder Faraday, Charles Farber, David Fardo, Samar Farha, Candace Farmer, Leanna Farnam, Diane Fatkin, Mary Feitosa, Adam Felsenfeld, Yuanqing Feng, Mike Feolo, Lindsay Fernandez-Rhodes, Nicole Ferraro, Jorge Ferrer, Kendra R. Ferrier, Jessica Fetterman, Camila Figueiredo, Tasha Fingerlin, Hilary Finucane, Annette Fitzpatrick, Jonathan Michael Flanagan, Jason Flannick, Katherine Fleck, Travis Fleming, Caitlin Floyd, James Floyd, Alison Fohner, Bernard Fongang, Myriam Fornage, Sarah Fortier, Ervin Fox, Nora Franceschini, Laurent Francioli, Alan Franklin, Chris Frazar, Jennifer French, Amanda Fretts, Mao Fu, Haoyi Fu, Yi-Ping Fu, Christian Fuchsberger, Stephanie M. Fullerton, Lucinda Fulton, Stacey Gabriel, Sarah Gagliano Taliun, Weiniu Gan, Santhi Ganesh, Andrea Ganna, Peter Ganz, Shouguo Gao, Xu Gao, Gao/Musong Gao, Christine Kim Garcia, Melissa Garcia, Michael Garrett, Melanie Garrett, Joseph Gasper, Nadine Gassner, William James Gauderman, Kyle Gaulton, Amadou Gaye, Sheila Gaynor, Brady Gaynor, Steven Gazal, Bruce Gelb, Xin Geng, Michele Gentili, Mark Geraci, Soren Germer, Robert Gerszten, Daniel Geschwind, Sina Gharib, Auyon J. Ghosh, Auyon Ghosh, Richard Gibbs, Steven Gilhool, Frank Gilliland, Madeline Gillman, Niles Gilmore, Stefanija Giric, Mark Gladwin, David Glahn, Joseph Glessner, LaShaunta Glover, Suneeta Godbole, Jennifer Godfrey Ponce, David Goff, Stephanie Gogarten, Ilana Goldberg, Rahul Gondalia, Dawei Gong, Hector Gonzalez, Mark Goodarzi, Matthew Goodman, Victor Gordeuk, Harald Goring, Daniel Gottlieb, Jérôme Goudet, Misa Graff, Jan Graffelman, Sarah Graham, Jacob Graham, Morgan Grams, Einat Granot-Hershkovitz, Meagan Grant, Penelope Graves, Sharon Graw, Kathryn J. Gray, Michael Griswold, Megan Grove, C. Charles Gu, Yongtao Guan, Weihua Guan, Miguel Guardado, Xiuqing Guo, Resmi Gupta, Namrata Gupta, Maria Gutierrez-Arcelus, Daniel E. Guzman, Edward Ha, Mary Haas, David M. Haas, Jeff Haessler, Georg Hahn, Yang Hai, Cassie Hajek, Hakon Hakonarson, Lauren Hale, Jennifer Halford, Michael Hall, Amelia Weber Hall, Ira Hall, Arda Halu, Anne Hamik, Michael Hammond, Bob Handsaker, Sue Hankinson, Sarah Hanks, Patrick Hanly, David Hanna, Nadia Hansel, Matthew Hansen, Manjit Hanspal, Luning Hao, Ross Hardison, Bernhard Haring, Laura Harrington, Daniel Harris, Kelley Harris, Alexandre Harris, Natalie Hasbani, David Haussler, Nicola L. Hawley, Lystra Hayden, Kathleen Hayden, Jiang He, Karen He, Nancy Heard-Costa, Ben Heavner, Susan Heckbert, Julian Hecker, Scott Heemann, Jonathan Brett Heimlich, Lacey Heinsberg, Ryan Hernandez, David Herrington, Craig Hersh, Bertha Hidalgo, Heather Highland, Blanca Himes, Joel Hirschhorn, Marie-France Hivert, James Hixson, Brian Hobbs, Chani Hodonsky, Chancellor Hohensee, John Hokanson, Yuling Hong, Charles Hong, Yun Soo Hong, Michael Honigberg, Martha Horike-Pyne, Steve Horvath, Karin Hoth, Lifang Hou, Candace M. Howard-Claudio, Tzu-Hung Hsiao, Chao (Agnes) Hsiung, Yi-Hsiang Hsu, Li Hsu, Sarah Hsu, Donglei Hu, Bo Hu, Jie Hu, Yao Hu, Xiaowei Hu, Tianxiao Huan, Meng Huang, Tianyi Huang, Alicia Huerta, Jennifer Huffman, Timothy Hughes, Daniel Hui, Scott Huntsman, Shih-Jen Hwang, Chii Min Hwu, Hae Kyung Im, Marguerite Ryan Irvin, Carmen Isasi, Jesse Islam, Yuval Itan, Kruthika Raman Iyer, Sidd Jaiswal, Yasminka A. Jakubek, Seonkyeong Jang, Cashell Jaquish, Gail Jarvik, Thomas Jaworek, Samantha Jensen, Paul Jensen, Alokkumar Jha, Xueqiu Jian, Min-Zhi Jiang [edit], Hongmei Jiang, Yu Jiang, Zhenghui Jiang, Jicai Jiang, Jin Jin, Sheng Chih Jin, Roby Joehanes, Eric Johanson, Jill Johnsen, Andrew Johnson, Randi K. Johnson, Lenora Johnson, Mari Johnson, Adam L. Johnson, Craig Johnson, Rich Johnston, Michelle Jones, Michelle Jones, Kimberly Jones, Jaehyun Joo, Daniel Jordan, Aparacio Jose, Paule Valery Joseph, Brian Joyce, Dan Ju, Renae Judy, Goo Jun, Sean Jurgens, Anne Justice, Priyadarshini Kachroo, Linda Kachuri, Ravi Kalhan, Jonathan Kaltman, Rita Kalyani, Debora Kamin Mukaz, Masahiro Kanai, Kanika Kanchan, Guolian Kang, Hyun Min Kang, Robert Kaplan, David Karasik, Sharon Kardia, Silva Kasela, Sekar Kathiresan, Yuriko Katsumata, Ronit Katz, Daniel H Katz, Joel Kaufman,

Katerina Kechris, Brendan Keenan, Rebecca Keener, Spencer Kelley, Rachel S. Kelly, Shannon Kelly, Tanika Kelly, Karl Kelsey, Eimear Kenny, Ali Keramati, Amena Keshawarz, Michael Kessler, Bryan Kestenbaum, Shareef Khalid, Alyna Khan, Emir Khatipov, Deepak Khatry, Amit Khera, Sumeet Khetarpal, Minjung Kho, Muin Khoury, Eric Kim, Woori Kim, Ellen Kim, Wonji Kim, Kyeezu Kim, Michelle Kim, Andy Kim, John Kim, Ryan Kim, Hyunju Kim, Eileen King, Greg Kinney, Krzysztof Kiryluk, Rick Kittles, Steven Kittner, Jorge Kizer, Malgorzata Klauzinska, Erica Kleinbrink, Robert Klemmer, Stacey Knight, Darae Ko, Barbara Konkle, Charles Kooperberg, Tal Korem, Jelena Kornej, Anna Kottgen, Christina Kourkoulis, Satoshi Koyama, Brian Kral, Holly Kramer, William Kraus, Mohanraj Krishnan, Akshaya Krishnaswamy, Harlan Krumholz, Laura Kubzansky, Rajesh Kumar, Allison Kupsco, Gulriz Kurban, Pui-Yan Kwok, Wassim Labaki, Roland Laboulaye, Sweta Ladwa, Alain Laederach, David Lafon, Chi Keung Lam, Jacqueline Lane, John Lane, Ethan Lange, Leslie Lange, Christoph Lange, Tuuli Lappalainen, Caleb Lareau, Martin Larson, Katie Larsson, Edward Lau, Lenore Launer, Cathy Laurie, Marie Lauzon, Brandon Lê, Suzanne Leal, Matthew Lebo, Meryl LeBoff, David Lederer, Seung-been Steven Lee, Wen-Jane Lee, I-Te Lee, Sanghun Lee, Christina Lee, Grace Lee, Sujin Lee, Dongwon Lee, Jonathon LeFaive, Claire Leiser, Rozenn Lemaitre, Samantha Lent, Petra Lenzini, Aaron Leong, Guillaume Lettre, Albert Levin, Daniel Levy, Joshua Lewis, Huiqing Li, Bingshan Li, Xingnan Li, Changwei Li, Yang Li, Taibo Li, Zilin Li, Jun Li, Xihao Li, Gang Li, Hongzhe Li, Jiang Li, Yun Li, Xiaohui Li, Jingjing Liang, Wil F. Lieberman-Cribbin, L. A. Liggett, Diane Lim, Elise Lim, Andrew Limper, Xihong Lin, Honghuang Lin, Danyu Lin, Bridget Mengshan Lin, Henry Lin, R. Coleman Lindsley, Elizabeth Litkowski, Amarise Little, Weixuan Liu, Yongmei Liu, Jiayan Liu, Simin Liu, Delong Liu, Ching-Ti Liu, Mengzhen Liu, Weifang Liu, Yu Liu, Dajiang Liu, Chunyu Liu, Xiaoming Liu, Eric Lock, Douglas Loesch, Stephanie London, Michelle Long, Ryan Longchamps, Will Longstreth, Ruth J.F. Loos, Camila Lopes-Ramos, Noah Lorincz-Comi, Michael Love, Shelly-Ann Love, Oswaldo Lozoya, Yingchang Lu, Yuan Lu, Steven Lubitz, Jessica Lundin, Kathryn Lunetta, Sheng Luo, Yang Luo, James Luo, Yuan Luo, Kai Luo, Pamela Lutsey, Sharon Lutz, Yanlin Ma, Jiantao Ma, Daniel MacArthur, Mitchell Machiela, Taralynn Mack, Purnema Madahar, Tracy Madsen, Ulysses Magalang, Anubha Mahajan, Michael Mahaney, Matthew Maher, Joe Maher, Arch (Chip) Mainous, Timothy Majarian, Angel CY Mak, Rajeev Malhotra, Nicholas Mancuso, Ani Manichaikul, Samantha Manna, Alisa Manning, Arjun Manrai, JoAnn Manson, Casey Marchek, Gregory Marcus, Lisa Martin, Fernando Martinez, Yessica Martinez, Susan Mathai, Rasika Mathias, Ravi Mathur, Scott Matson, Richard Mayeux, Diego R. Mazzotti, Patrick McArdle, Sean McCabe, Steve McCarroll, Mark McCarthy, Merry-Lynn McDonald, Barbara McDonough, Stephen McGarvey, Claire McGroder, Barbara McKnight, John McLenithan, David McManus, Coleen Mcnamara, Becky McNeil, Michael McQuillan, Tonya McSherry, Hao Mei, James Meigs, George Mensah, Josep Mercader, Tony Merriman, Geralyn Messerlian, Luisa Mestroni, Ginger Metcalf, Mariah Meyer, Deborah A Meyers, Michael Mi, Xinlei Mi, Karen Miga, Anna Mikhaylova, Julie Mikulla, Amy Miller, Clint Miller, Danny Miller, Christopher Miller, Nancy Min, Mollie Minear, Ryan L Minster, Biswapriya Misra, Braxton D. Mitchell, Patrick Mitchell, Gary F. Mitchell, Miremad Moafi-Madani, Deborah Moeller, Sanghamitra Mohanty, Karen Mohlke, Matt Moll, Jean Monlong, Rebecca Montalvan, Arthur Montanari, May E. Montasser, Courtney Montgomery, Stephen Montgomery, Kyung Moon, Jee-Young Moon, Vamsi Mootha, Andrew Moran, Valerie Morrill, Andrew Morris, Alanna C Morrison, Jarrett Morrow, Maria Isabel Moscardó García, Arden Moscati, Thomas Mosley, Jonathan Mosley, Kristine Movalli, Veronica Mungai, Joanne Murabito, Jamie Murkey, Venkatesh Murthy, Shaila Musharoff, Rajeeva Lochan Musunuri, Donna Muzny, Josyf C Mychaleckyj, Jonathan Na, Girish Nadkarni, Abhishek Nag, Pavithra Nagarajan, Rakhi Naik, Tetsushi Nakao, Hongmei Nan, Drew Nannini, Vivek Naranbhai, Giuseppe Narzisi, Rami Nassir, Pradeep Natarajan, Anjali Nath, Victor Nauffal, Ana Navas-Acien, Matt Nayor, Benjamin Neale, Sergei Nekhai, Sarah C. Nelson, Cheryl Nelson, Christopher Newton-Cheh, Maggie Ng, Debby Ngo, Marilyn Ngo, Lan Nguyen, Jayna Nicholas, Jovia Nierenberg, Faye Norby, Kari North, Seyed Mehdi Nouraie,

Adam Novak, Paul Nyquist, Grace O'Brien, Jeff O'Connell, Tim O'Connor, Brian O'Connor, George O'Connor, Christopher O'Donnell, Wanda O'Neal, Christopher O'Sullivan, Heather Ochs-Balcom, Franklin Ockerman, Elizabeth Oelsner, Take Ogawa, Sam Oh, Young S. Oh, Paul Chukwuebuka Okoro, Michael Olivier, Nels Olson, Jean L. Olson, Jasmine M. Olvany, Hiromi Ono, Richard Oppong, Peter Orchard, Victor Ortega, Kevin Osborn, Jim Ostell, Jacqueline Otto, Demetria Pace, Betty Sue Pace, Allan Pack, Eric Padron, Grier Page, David T. Paik, Helena Palma Gudiel, Nicholette Palmer, Nathalie Pamir, Yang Pan, James Pankow, Nathan Pankratz, Daniel Panyard, George Papanicolaou, Vibhu Parcha, Yoson Park, Nguyen Park, DFAAPA,, Stephen Parker, Alyssa Parker, Margaret Parker, Kaavya Paruchuri, Chirag Patel, Sanjay Patel, Jay Patel, Roshni Patel, Aniruddh Patel, Ravi Patel, Benedict Paten, Candace Patterson, Anna Louise Peljto, Gina Peloso, Victoria Pemberton, Juan Manuel Peralta, Alexandre Pereira, Marco Perez, James Perry, Andrew Perry, Ulrike Peters, Tess Peterson, Mary Pettinger, Patricia Peyser, Nathan Pezant, Lawrence S Phillips, Grace Pien, Maria Pino-Yanes, Shraddha Piparia, James Pirruccello, Achilleas Pitsillides, John Platig, Alexander Platt, Mary Playdon, Anna Podolanczuk, Craig Pohl, Hannah Poisner, Linda Polfus, Maria Politis, Toni Pollin, Francesca Polverino, Bianca Porneala, Wendy Post, Tess D. Pottinger, Julia Powers Becker, Diddier Prada, Katherine Pratte, Meher Preethi Boorgula, Michael Preuss, Alkes Price, Jonathan Pritchard, Anna Prizment, Dmitry Prokopenko, Mike Province, Bruce Psaty, Clive Pullinger, Shaun Purcell, Sandra Purves, Hira Qadir, Pankaj Qasba, Qibin Qi, Yong Qian, Huijun Qian, Chen’ao Qian, Dandi Qiao, Zhaohui Qin, Huiqi Qu, Gloria Quach, Corbin Quick, Cristina Rabadan-Diehl, Daniel Rader, Nicholas Rafaels, Leslie Raffel, Laura Raffield, Dan Raftery, Sridharan Raghavan, Mohammad H. Rahbar, Vasan S. Ramachandran, D.C. Rao, Prashant Rao, Sara Rashkin, Laura Rasmussen-Torvik, Aakrosh Ratan, Mitali Ray, Debashree Ray, Soumya Raychaudhuri, Susan Redline, Robert Reed, Sarah Reese, Elizabeth Regan, Jalees Rehman, Patrick F. Reilly, Christopher Reilly, Amy D. Reily, Alex Reiner, Kathryn Rexrode, Lindsay Reynolds, Ken Rice, Stephen Rich, Michiel Rienstra, Nicolas Robine, Cassianne Robinson-Cohen, Alison Rocco, Angela Rock, Dan Roden, Kameron Rodrigues, Annabelle Rodriguez, Veronique Roger, Kathryn (Kaye) Roll, Mary Rooney, Jonathan Rosand, Carolina Roselli, Yohei Rosen, Jerome Rotter, Peifeng Ruan, Ingo Ruczinski, Michael Rueschman, Emily Russell, Sarah Ruuska, Kathleen Ryan, Min Hyung Ryu, Wael Saber, Ester Cerdeira Sabino, Aniko Sabo, Nancy Saccone, Vandana Sachdev, Aabida Saferali, Sandra E. Safo, Shivani Sahni, Phuwanat Sakornsakolpat, Sandra Salazar, Danish Saleheen, Rany Salem, Shabnam Salimi, Steven Salzberg, Mark Sanborn, Kevin Sandow, Dharambir Sanghera, Vijay G. Sankaran, Muralidharan Sargurupremraj, Chloé Sarnowski, Claudia Satizabal, Gretchen Saunders, Richa Saxena, Ben Scammell, Gerard Schellenberg, Pascal Schlosser, Peter Schnatz, Jenny Schoenberg, Charlene Schramm, Daniel Schrider, Karen Schwander, David Schwartz, Chip Schwartz, Marvin Schwarz, Frank Sciurba, Fritz Sedlazeck, Mark Segal, Max A. Seibold, Jonathan Seidman, Christine Seidman, Peggy Sekula, Margaret Sunitha Selvaraj, Elizabeth Selvin, Jungkyun Seo, Minseok Seo, Vladimir Seplyarskiy, Norma Serrano Diaz, Sudha Seshadri, Magdalena Sevilla Gonzalez, Amanda Seyerle, Mahsima Shabani, Lincoln Shade, Aladdin Shadyab, Christian Shaffer, Palak Shah, Binal Shah, Ravi Shah, Sanjiv Shah, Amitabh Sharma, Andrew Sharp, Jessica R Shaw, Vivien Sheehan, Elizabeth Sheets, Christopher John Sheldahl, Yufeng Shen, Stephanie L. Sherman, Steve Sherry, Amol Shetty, Wayne Hui-Heng Sheu, Kevin Shianna, Daichi Shimbo, M. Benjamin Shoemaker, Meghan I Short, Katherine Shutta, Yichen Si, Chris Siege, Brian Silver, Allison Silverman, Edwin Silverman, Jeannette Simino, Noah Simon, Mortiz Sinner, Nasa Sinnott Armstrong, Colleen Sitlani, Robert Sladek, Patrick Sleiman, Craig Smail, Gustav Smith, Jennifer Smith, Albert Vernon Smith, Nicholas Smith, Ashley Smith, Josh Smith, Benjamin Smith, Sylvia Smoller, Linda Snetselaar, Soren Snitker, Beverly Snively, Lucia Sobrin, Rachel Soemedi, Tamar Sofer, Mashaal Sohail, Sophie Sokolow, Yun S. Song, Wei Song, Nona Sotoodehnia, Melissa Spear, David Spies, Simon Spivack, Cassie Spracklen, Vinodh Srinivasasainagendra, Deepak

Srivastava, Lauren Stalbow, Maggie Stanislawski, Jason Stein, David Steinberg, Debra Stern, James Stewart, Adrienne M. Stilp, Adam Stine, Nathan Stitziel, Garrett Storm, Elizabeth Streeten, Kathleen A. Stringer, Anne Sturcke, Jessica Lasky Su, Shakira Suglia, Patrick Sullivan, Arvis Sulovari, Pavel Sumazin, Carly Summarell, Steven Sun, Xiao Sun, Han Kevin Sun, Quan Sun, Haoqi Sun, Zequn Sun, Albert Sun, Aishwarya Sundaresan, Yun Ju Sung, Shamil R Sunyaev, Ida Surakka, Alicia Sutherland, Mindy Szeto, Zachary Szpiech, Adam Szpiro, Carole Sztalryd, Fred K. Tabung, Usman Tahir, Soheyla Taie-Tehrani, Hua Tang, Yaling Tang, Weihong Tang, Ran Tao, Margaret Taub, James G. Taylor, Simeon Taylor, Kent D. Taylor, Matthew Taylor, Bamidele Tayo, Cynthia Tchio, Marilyn Telen, Larisa Tereshchenko, James Gregory Terry, Florian Thibord, Alastair Thomson, Timothy A. Thornton, Machiko Threlkeld, Bharat Thyagarajan, Phyllis Tien, Adrienne Tin, Lesley Tinker, David Tirschwell, Sarah Tishkoff, Hemant Tiwari, Deirdre Tobias, Dominic Tong, Catherine Tong, Russell Tracy, Mark Trinder, Martin Tristani, Michael Tsai, Ming-Ju Tsai, Connie Tsao, Serena Tucci, Ernest Turro, Daniel Unger, Sarah Urbut, Dhananjay Vaidya, Eric Van Buren, David Van Den Berg, Lisa VanWagner, Jose Vargas, Marie Verbanck, Karine A. Viaud Martinez, Peter Visscher, Caitlyn Vlasschaert, Joanna von Berg, Ann Von Holle, Scott Vrieze, Marijana Vujkovic, Robin Wagner, Michael Wagner, Jason Waligorski, Tarik Walker, Maura E. Walker, Kaneesha Wallace, Robert Wallace, Matthew Walter, Avram Walts, Emily Wan, Cuicui Wang, Zhe Wang, Yanbing Wang, Xin-An Wang, Yuxuan Wang, Zhaoming Wang, Penglong Wang, Biqi Becky Wang, Ningyuan Wang, Jiongming Wang, Heming Wang, Tao Wang, Rachel A. Warren, Karol Watson, Reem Waziry, Daniel E. Weeks, Lachelle Weeks, Gina Wei, Joshua Weinstock, Bruce Weir, Scott T Weiss, Jia Wen, Fayuan Wen, Christine Wendt, Lu-Chen Weng, Ellen Werner, Jennifer Wessel, Kenneth Westerman, Marsha Wheeler, Heather Wheeler, Seamus Whelton, Wendy White, Nedra Whitehead, Eric Whitsel, Kerri L. Wiggins, John Wilkins, Charles Williams, Scott Williams, L. Keoki Williams, Jessica Williams-Nguyen, Steffanie Wilson, James Wilson, Carla Wilson, Ava Wilson, Lara Winterkorn, Karen Winters, Mary Wojczynski, Brooke Wolford, Hyejung Won, Ambroise Wonkam, Alexis Wood, Prescott Woodruff, Baojun Wu, Haodi Wu, Qing Wu, Peitao Wu, Lang Wu, Joseph Wu, Ann Wu, Kristin Wuichet, Mark Wurfel, Vanessa Xanthakis, Lluvia Xia, Shujie Xiao, Chunlin Xiao, Zihao Xin, Hanfei Xu, Weiling Xu, Lingyun Xu, Huichun Xu, Ye Yan, Qi Yan, Lisa Yanek, Stephanie Yang, Chaojie Yang, Qiong Yang, Yu-Chung Yang, Ivana Yang, Yuichiro Yano, Jie Yao, Toni-Ann Yapp, Ronit Yarden, Eugene Yaschenko, Mohammad Yaser, Michelle Yau, Jane Ye, Xianyong Yin, Zachary Yoneda, Kimberley Youkhana, Tracey Young, Erica Young, Kristin Young, Bessie Young, Michael Young, Kendra Young, Marston Youngblood, Zhi Yu, Miao Yu, Fangtang Yu, Bing Yu, Fiona Yuen, Jeong Yun, Norann Zaghloul, Anthony Zannas, Habil Zare, Joe Zein, Seyedeh Maryam Zekavat, Xiaohua Douglas Zhang, Wei Zhang, Yingze Zhang, Daiwei Zhang, Yu-Hang Zhang, Jerry Z. Zhang, Yixin Zhang, Jingzhou Zhang, Xiaoyu Zhang, Xu Zhang, Grace Man Zhang, Sai Zhang, Xinruo Zhang, Chao Zhang, Ying Zhang, Lue Ping Zhao, Xutong Zhao, Xuefang Zhao, Wei Zhao, Yinan Zheng, Degui Zhi, Ying Zhou, Hufeng Zhou, Laura Zhou, Beiyan Zhou, Jiayan Zhou, Xiang Zhou, Luke Zhu, Xiaofeng Zhu, Hongtu Zhu, Wenjuan Zhu, Yonghua Zhuang, Elad Ziv, Michael Zody, Sebastian Zoellner, Barry Zorman, Assistants:, , Norma Aguilera, Ameena Al-Amin, Daniel Barrett, Marie Barth, Susanne Bartlett, Erica Bertisch, Trevor Bierig, Lisa Billington, Dranette Branson, Lynn Brazil, Camille Breaux, Tonia Brown, Pamela Burton, Felecia Campbell, Sabrina Carrington, Grace Choi, Sarah Katherine Cody, Kathy Colbert, Brigidann Cooper, Rhea Cosentino, Phyllis Crockett, Ashley Dantzler, Isabel Davis, Jennifer Dean, Maria Rizzo DePaoli, Jennifer Elhawary, Kelley Foyil, Yvonne Fraser, Amber Gist, Jung Ho Gong, Gisselle Gonzalez, Daniel Greene, Carla Hammond, AJ Hirsch Allen, Kuan-Yi Hung, Consuelo Kleemann, Iain Konigsberg, Tammy Markus, Deborah McDonald, Alexis Michal, Lauren Mineo, Enrique Pena, Dina Pontarelli, Zoe Poyen, Yugandi Ranaweera, Catherine Reeves, Katia Rodriguez, Ilanna Rosario, Leshana Saint-Jean, Bonnie L Schoenbein, Kristy Smith, Jeremy Straughn, Randi

Sullivan, Jason Thompson, Shelby Thompson, Daune Thorington, Stacie Truszkowski, Rose Vallines, Ann Walsh, Peggy White, Hannah Whitley, Ann Whitney, Sandy Zellner

TOPMed Analysis Working Group

Gonçalo Abecasis, Rafet Al-Tobasei, Terri Beaty, Takis Benos, Larry Bielak, Joshua Bis, Thomas Blackwell, John Blangero, Victor Borda, Jennifer Brody, Brian Browning, Deepika Burkardt, Jenna Carlson, Han Chen, So Mi Cho, Michael Cho, Seung Hoan Choi, Ren-Hua Chung, Matthew Conomos, Brandon Coombes, L. Adrienne Cupples, Michelle Daya, Mariza de Andrade, Xutao Deng, Jasmin Divers, Ron Do, Shiron Drusinsky, Josée Dupuis, Amanda Elliott, David Fardo, Jessica Fetterman, Hilary Finucane, Caitlin Floyd, Nora Franceschini, Jennifer French, Weiniu Gan, Soren Germer, Stephanie Gogarten, Harald Goring, Jan Graffelman, Xiuqing Guo, Mary Haas, David Haussler, Ben Heavner, Scott Heemann, Ryan Hernandez, Blanca Himes, Tzu-Hung Hsiao, Sarah Hsu, Donglei Hu, Yao Hu, Jennifer Huffman, Scott Huntsman, Hae Kyung Im, Kruthika Raman Iyer, Jicai Jiang, Rich Johnston, Masahiro Kanai, Hyun Min Kang, Sharon Kardia, Tanika Kelly, Ali Keramati, Michelle Kim, Charles Kooperberg, Anna Kottgen, Satoshi Koyama, Roland Laboulaye, Ethan Lange, Cathy Laurie, Yun Li, Xihong Lin, Danyu Lin, Delong Liu, Ching-Ti Liu, Douglas Loesch, Yingchang Lu, Jessica Lundin, Kathryn Lunetta, Daniel MacArthur, Timothy Majarian, Angel CY Mak, Ani Manichaikul, Alisa Manning, Casey Marchek, Rasika Mathias, Sean McCabe, Merry-Lynn McDonald, Barbara McKnight, Julie Mikulla, Amy Miller, Ryan L Minster, Braxton D. Mitchell, May E. Montasser, Valerie Morrill, Andrew Morris, Pradeep Natarajan, Benjamin Neale, Maggie Ng, Jeff O'Connell, Tim O'Connor, Grier Page, Nicholette Palmer, George Papanicolaou, Benedict Paten, Gina Peloso, James Perry, Patricia Peyser, Dmitry Prokopenko, Mike Province, Bruce Psaty, Pankaj Qasba, Dandi Qiao, Laura Raffield, Alex Reiner, Ken Rice, Ingo Ruczinski, Kathleen Ryan, Frank Sciurba, Laura Scott, Margaret Sunitha Selvaraj, Magdalena Sevilla Gonzalez, Amanda Seyerle, Colleen Sitlani, Craig Smail, Albert Vernon Smith, Jennifer Smith, Josh Smith, Tamar Sofer, Vinodh Srinivasasainagendra, Adrienne M. Stilp, Jessica Lasky Su, Xiao Sun, Zachary Szpiech, Hua Tang, Kent D. Taylor, Timothy A. Thornton, Hemant Tiwari, Dominic Tong, Md Mesbah Uddin, Eric Van Buren, Karine A. Viaud Martinez, Tao Wang, Daniel E. Weeks, Bruce Weir, Jennifer Wessel, Kenneth Westerman, Shujie Xiao, Huichun Xu, Lisa Yanek, Zhi Yu, Seyedeh Maryam Zekavat, Wei Zhao, Degui Zhi, Hufeng Zhou, Ying Zhou, Xiaofeng Zhu, Michael Zody, Sebastian Zoellner, Trevor Bierig, Lynn Brazil, Brigidann Cooper, Rhea Cosentino, Yvonne Fraser, Carla Hammond, Catherine Reeves, Kristy Smith, Jeremy Straughn, Daune Thorington
